# Demographic Differences in Compliance with COVID-19 Vaccination Timing and Completion Guidelines in the United States

**DOI:** 10.3390/vaccines11020369

**Published:** 2023-02-06

**Authors:** Peiyao Zhu, Victoria Zhang, Abram L. Wagner

**Affiliations:** Department of Epidemiology, School of Public Health, University of Michigan, 1415 Washington Heights, Ann Arbor, MI 48109, USA

**Keywords:** COVID-19 vaccine, vaccination coverage, vaccination compliance, race, religion, political affiliation

## Abstract

Background: The development of vaccines has been a significant factor in eliminating the pandemic caused by the novel coronavirus (SARS-CoV-2). However, the primary series vaccination rate still falls short of our expectations, with an even lower rate of uptake for booster shots. This study examined demographic patterns of COVID-19 vaccination compliance by assessing patterns in the timing of the vaccine series start and vaccination completion and characterizing people by compliance with vaccination recommendations. Methods: A cross-sectional survey was conducted online in August 2022. Participants answered questions about the COVID-19 vaccine and questions related to their personal backgrounds. We assessed the impact of demographic factors on COVID-19 vaccination using multivariable regression modeling. Results: Among 700 eligible participants, 61% (389) were highly adherent (i.e., started by late 2020 and received a booster dose), 22% (184) were moderately adherent (i.e., started later than June 2021, and/or did not receive the booster dose), and 17% (127) were unvaccinated. Compliance was relatively low among non-Hispanic Black Americans, those with no religious affiliation, and among Independents and Republicans. Conclusion: Vaccination compliance varies across demographic groups. Race/ethnicity, religion, and political affiliation are highly associated with vaccination compliance. To promote vaccination compliance and decrease vaccine hesitancy, the government and healthcare institutions should establish a positive image to obtain public trust and adopt effective vaccine education and intervention.

## 1. Introduction

The new Coronavirus (SARS-CoV-2) has been responsible for around 635 million confirmed cases and 6.61 million deaths since its emergence in December 2019 in Wuhan, China [1]. Governments have actively endeavored to stop the spread of the pandemic by providing incentives for vaccination. Vaccines have been used as successful interventions and preventative measures in large-scale outbreaks throughout history, reducing the transmissibility and mortality of infectious diseases. According to the CDC report in 2016, nine routine vaccines for kids have significantly decreased the incidence of diseases by more than 90%, saving $13.5 billion in direct expenses and $68.8 billion in total societal costs [2,3]. Vaccines not only improve an individual’s health by reducing the risk of illness but can have societal benefits by reducing the need for a family to care for cases and through herd immunity, which occurs when enough of the population is disease-resistant to limit the likelihood that an infected person will come into contact with a susceptible person [4,5]. Therefore, vaccines have proven to be of great importance in safeguarding public health as well as reducing the economic burden as long as populations have high adherence to recommendations about vaccine timing and dose completion. 

In the development and deployment of the COVID-19 vaccine, multiple phase III trials have demonstrated its effectiveness against severe acute respiratory syndrome Coronavirus 2 (SARS-CoV-2) infection [6]. However, booster doses are necessary to maintain the vaccine’s efficacy against the virus and ensure its continued effectiveness in mitigating the spread of the disease. Several studies reported that the effectiveness of vaccines decreases over time with the emergence of different COVID-19 variants [7,8,9]. Two doses of the primary vaccine have limited resistance to variants such as delta and omicron. One study in New York found that as the delta form predominated, the three vaccines’ efficacy against COVID-19 decreased while their ability to prevent hospitalization remained high [7]. When comparing individuals who received their vaccination in January 2021 and those who received their vaccination in April 2021, the increased risk for infection was 2.26-fold [8]. Besides, getting a booster is important in order to increase protection, but this protection wanes over time since the serum antibody levels naturally decline after a few months post-vaccination [9]. However, another problem arose: despite the fact that vaccines are free, some individuals were hesitant to get vaccinated or receive booster doses. According to the US CDC COVID-19 data report, only 68.8% of the US population completed the primary vaccination series, with even fewer people receiving booster doses (Population ≥ 18 years of age = 14.7%, ≥65 years of age = 32.6%) [10].

The timely development of the vaccine as an effective long-term method to prevent the virus’s quick spread gave people hope of overcoming the pandemic. However, due to production, distribution, and storage issues, the vaccine was initially only accessible to high-risk populations such as healthcare physicians, first responders, and vulnerable individuals with immune system impairments. Even though these problems were solved by the government’s valid consolidation of resources, the imbalance between supply and demand affected the population’s intention to get vaccinated. According to previous research, the scarcity of the COVID-19 vaccine reduced people’s willingness to get vaccinated because of a decrease in their perceived priority of being vaccinated [11]. Since May 2021, the supply of vaccines has exceeded demand, as exemplified by stagnating vaccination rates [12]. Therefore, vaccine timing is linked to vaccine supply and demand. To ensure expeditious vaccination coverage and herd immunization, it is essential to maintain a sufficient supply of vaccines and increase the number of vaccination sites.

Differences in attitudes toward vaccination across racial, political, and religious groups have arisen over the COVID-19 pandemic in the United States. From a sociological standpoint, some empirical research indicated that people’s ideas and worldviews have a significant impact on how they perceive and accept risk [13]. Vaccines, an innovation, are perceived as a risk by an individual or other unit of adoption. Thus, individuals’ behavior regarding whether to vaccinate as a risk can be influenced by their personal experiences and worldview (including religion), social background (such as race/ethnicity), and their political views. According to the most recent CDC report, non-Hispanic (NH) Black Americans have a lower primary series completion rate of 44.2% and a booster rate of only 6.7% compared to other racial and ethnic groups [10]. By political affiliation, Republicans have less trust in COVID-19 vaccines than Democrats [14]. Related research found that a 76% higher excess death rate was observed in Republicans than Democrats at the individual level during the initial years of the COVID-19 pandemic [15]. Additionally, research findings have suggested that both race/ethnicity and religion should be taken into consideration when establishing communications and promotional strategies regarding vaccination programs [16].

Vaccine uptake has stalled in the United States. In light of continued challenges in increasing vaccine uptake, it is important to have a more in-depth analysis of the trajectory of vaccine coverage and what differences exist across various sociodemographic groups. Previous literature has explained the significant role of the historical, political, and socio-cultural context in individual decision-making about vaccination, so it is necessary for us to identify the population characteristics of low vaccine compliance by examining these differences. [17] In addition, according to studies of previous outbreaks, understanding vaccination attitudes based on various demographic factors is crucial for developing current and future epidemic response policies and communication techniques. Thus, we aimed to systematically examine demographic patterns of compliance with COVID-19 vaccination and mainly explore how race/ethnicity, religion, and political affiliation impact the COVID-19 vaccination. In specific, (1) to assess the pattern in vaccination completeness, (2) to assess the pattern in the timing of vaccination start, and (3) to characterize individuals by compliance with vaccination guidelines (combining completeness and time). 

## 2. Materials and Methods

### 2.1. Study Population

Dynata, a survey research company, was responsible for recruiting participants through social media and other advertising. US adults included in their sampling frame were eligible for inclusion in this study. An age-sex nested quota system was adapted in our model; a certain number of people by female/male gender and in six age groups (18–24, 25–34, 35–44, 45 and up) were purposefully included in the dataset. We created sample weights so that our study population reflected national estimates from the US Census in terms of age, gender, region of the country, education, and race/ethnicity. Our sample size calculation was based on an aim for a previous project—to precisely estimate the proportion of individuals vaccinated at a given time; with a sample size of 800, we could estimate an outcome proportion of 50% (a statistically conservative estimate), based on a margin of error of 4%, an alpha of 0.05, and a power of 80%.

### 2.2. Derived Variables

Participants filled out the survey themselves online. This study has three outcomes: vaccination status, series timing, and vaccine compliance. The vaccine questions were based on the Kaiser Family Foundation COVID-19 Vaccine Monitor. Vaccine status and series timing were derived from our questionnaire. According to participants’ self-reported responses, vaccination statuses were categorized into four groups. (1) unvaccinated; (2) partially vaccinated; (3) people who received one dose of Johnson & Johnson or two doses of other vaccine brands are considered to have completed the vaccination; (4) those who received more than one dose of Johnson & Johnson or more than two doses of other vaccine brands are deemed to have completed the booster. The series timing contained five groups based on the administration of the first dose: (1) unvaccinated; (2) no recall; (3) started in 2020 or early 2021; (4) started in late 2021; and (5) started in 2022.

Vaccine compliance is a combination of vaccination status and series timing. Unvaccinated groups were considered to have low compliance. The high compliance group refers to those who have started to receive the vaccine before July 2021 and have received at least one booster dose. The rest of the study population who received the vaccine later than June 2021 or did not receive the booster dose were classified as having moderate compliance.

Independent variables, including age, gender, race/ethnicity, political affiliation, education level, and region, were derived from the questionnaire. Race/ethnicity groups were condensed into NH White Americans, NH Black Americans, Hispanics, and others in consideration of sample sizes. Participants were asked whether they were Hispanic and about their racial heritage (with multiple options available). Those who chose more than one option were then asked which option best represented their racial backgrounds. We asked about religions using guidelines from the Pew Research Center [18]. The main religions categories in this study were Catholic/Orthodox, Evangelical, Jewish, Mainline, Muslim, Other Christian, and No religion. Buddhism, Hinduism, Islam, and Judaism were categorized under “Other religions.” Gender identity was determined using standards established by the American Association of Public Opinion Researchers [19].

### 2.3. Statistical Analysis

Demographic characteristics were presented as counts and proportions for statistical description. Sample weight was created using variables age, gender, region of the country, education, and race/ethnicity. The chi-square test was utilized to determine the statistical significance of the relationship between demographic variables and vaccine completion, series timing separately. Fisher’s exact test was used for gender and religion due to the limited sample size leading to low cell counts. 

The association between demographic variables and vaccine compliance was explored using a multivariable, multinomial logistic regression analysis with an odds ratio and 95% confidence interval to express the strength of the association. Medium and high COVID-19 vaccine compliance were compared against the base of low vaccination compliance. All data were analyzed in SAS version 9.4.

## 3. Results

Our survey received 806 valid responses: 751 respondents agreed to the informed consent, and 715 respondents took adequate time to complete the survey, with a response duration of more than 3 min. In addition, 700 (87% of valid responses) of them answered the majority of the survey’s demographic questions.

### 3.1. Vaccination Completion

Overall, 17% (127) had not been vaccinated, 5% (45) had received one dose of COVID-19 but had not completed the primary series, 15% (130) had completed the primary series but had not received any booster dose, and 63% (385) had received at least one booster dose.

*Race/ethnicity*. Table 1 shows the relationship between demographic groups and vaccination completion. There was a significant difference by race/ethnicity (*p* < 0.0001); the highest proportion of booster vaccine receipt was among NH White participants (70%) and was relatively low among NH Black participants (35%). Similarly, rates of non-vaccination were highest among NH Black participants (41%) and relatively low among NH White (16%) and other (11%) participants.

*Religion*. Religion also made a significant difference (*p* < 0.0001); The proportion of participants who had at least one booster shot was highest among Mainline participants (85%) and was relatively low among participants who identified as non-religious (52%) or with other religions (52%). Accordingly, non-vaccination rates were highest among none (28%) and other religion participants (34%), while Mainline Christian (7%) and Muslim (0%) participants had the lowest non-vaccination rates.

*Political affiliation*. Vaccination completion varied significantly across stated political affiliations (*p* = *0*.0018). Those identifying as Democrats had the highest proportion of getting booster shots (71%). In comparison, the Independents had a lower percentage (56%), and the Republicans had a slightly higher proportion (59%) than the Independent Party. Conversely, the Independents had the highest non-vaccination rate (27%), and the Democrats had a relatively low non-vaccination rate (11%).

*Other demographic variables*. Vaccination completion also varied significantly by age (*p* < 0.0001), gender (*p* = 0.0076), and education (*p* < 0.0001). Higher rates of receiving booster doses were associated with older age groups (73%). Males (66%) were more likely than females (60%) and other gender identity groups (20%) to complete the primary series and get booster shots. People with a bachelor’s degree had the highest booster dose vaccination rate of any degree level (79%), while people with a high school diploma or a lower degree had the highest non-vaccination rate and the lowest booster dose rate (49%).

### 3.2. Vaccination Timing

According to Table 2, 65% (423) of the participants began COVID-19 vaccination in 2020 or early 2021, with fewer starting in late 2021 (11%) and 2022 (2%). 

*Race/ethnicity*. There was a significant difference by race/ethnicity (*p* < 0.0001). Other races (70%) and NH White Americans (69%) had the highest proportion of beginning vaccination in 2020 or early 2021, and the early vaccination rate was lower among NH Black Americans (44%).

*Religion*. Religion was significantly associated with vaccine timing. Mainline (80%) and Muslim (80%) groups had the highest proportion of starting vaccinations early in 2020 or early 2021. Other religions (34%) and people who do not have religion (58%) had a relatively lower proportion of people starting vaccination in 2020 or early 2021, which corresponded with previous results that showed a higher booster vaccination rate in Mainline and a lower rate in other and non-religious groups.

*Political affiliation*. Political affiliation was significantly related to vaccine timing. In total, 72% of Democrats started vaccination in 2020 or early 2021, compared to 54% of Independents and 67% of Republicans.

*Other demographic variables*. Age (*p* < 0.0003), gender (*p* < 0.0104), and education (*p* < 0.0001) all had a significant association with vaccination timing. The highest proportion of early vaccination was among the older age group (72%), and it was relatively low among the 18–24 age group (47%). Compared to females (61%) and other gender identity groups (66%), males (69%) were more likely to start vaccination early, in 2020 or early 2021. Higher levels of education were associated with earlier COVID-19 vaccination timing. 74% of those with a bachelor’s or higher education level began vaccinations in 2020 and early 2021.

### 3.3. Vaccination Compliance

Based on vaccination completion and timing, 61% (389) were highly adherent (i.e., started by June 2020 and received a booster dose), 22% (184) were moderately adherent (i.e., started later than June 2021 or did not receive the booster dose), and 17% (127) were unvaccinated (Table 3).

In a model adjusted for all shown variables, we observed significant trends in compliance by race/ethnicity, religion, and political affiliation. By race/ethnicity, NH Black Americans had lower odds of medium (OR: 0.3, 95% CI: 0.1, 0.9) or high (OR: 0.2, 95% CI: 0.1, 0.5) compliance with COVID vaccination guidelines compared to NH White Americans. Conversely, Hispanic Americans had higher odds of medium compliance (OR: 3.1, 95% CI: 1.3, 7.0) but similar odds of high compliance compared to NH White Americans. By religion, medium or high compliance was relatively low among those with no religious affiliation compared to other groups. For political affiliation, both Independents and Republicans had lower odds of both medium and high compliance with COVID-19 vaccination compared to Democrats.

## 4. Discussion

Our study investigated demographic patterns of COVID-19 vaccination compliance, particularly at the time of vaccination timing and completion. Since the beginning of the pandemic, there have been concerns about how vaccination coverage could be relatively low in certain groups [15,16], which could lead to pockets of susceptibility to future outbreaks of disease. We surveyed the US adults among a variety of demographically varied groups. This study reveals significant differences in vaccination compliance, in terms of timing and series completion of COVID-19 vaccines, by race, religion, and political affiliation. 

*Race/Ethnicity*. In our study population, we found complex patterns of vaccination by demographic group. For instance, NH Black participants consistently had lower compliance compared to NH White participants. However, comparing Hispanic participants to their non-Hispanic white counterparts, they had higher odds of medium compliance but similar odds of high compliance. It can also be reflected in Table 1 that Hispanic participants had lower rates of non-vaccination but relatively high rates of not completing the vaccine series. Similar results were reported in other studies, showing that the majority of states had racial disparities in COVID-19 vaccination rates for both Black and Hispanic populations. Of the 45 states, 43 showed a Black-to-White disparity in COVID-19 vaccination rate, while 38 of the 42 states showed a Hispanic-to-White disparity [20]. The level of structural racism across states explains much of this disparity between states. Related research showed that differences in the severity of the observed structural disparities in COVID-19 vaccination for both the Black and Hispanic populations are substantially associated with structural racism, which presented as the unequal rollout of vaccination sites, unable to take time off work, lack of transportation, etc. [20,21]. Also, structural racism motivated chain reaction exacerbated vaccine compliance gaps. For example, vaccine hesitancy resulting from mistrust of healthcare providers and the government could be the following reason for racism to explain the racial difference in vaccine compliance. Research conducted in Detroit indicated that if NH Black Americans had similar confidence levels in healthcare providers as NH White Americans, 23% of the gap in vaccination uptake could be eliminated. A similar finding was reflected in the level of trust in government between Black and White Detroiters [22]. Structured racism significantly impacts health education programs that aim to increase patient trust in the medical system. Building trust in the government and the healthcare system would be challenging if structural racism cannot be eradicated [23].

*Religion*. Research also indicated an interesting finding that religion is significantly associated with vaccination completion and timing, while statistical significance does not maintain regarding vaccination compliance in subgroups of religion. Evangelicals and other religious groups had higher odds of medium vaccination compliance but lower odds of high vaccination compliance than Catholics/Orthodox. However, non-religious groups had consistently low odds of vaccination compliance. In a study in Australia, local areas with higher percentages of the population who do not identify with formal religions (i.e., no religion) tended to have lower vaccination rates [24]. One possible explanation is that, unlike religious people who adhere to or are influenced by dogma, people who do not identify with main religions may believe in spiritual and metaphysical concepts that lie outside religions [25]. Therefore, they are more sensitive to their own impressions and susceptible to societal opinions from social media or the surrounding environment. Alternatively, to put it another way, they are probably more prone to conspiracy beliefs or anti-vaccine sentiment. However, we observed the tremendous influence of vaccine conspiracy beliefs on people who are not only limited to non-religious people. Robertson et al. argued that religion had become a way for conspiracy theories to be normalized through their conception of shared similarities between conspiracy theories and religion (i.e., both holding beliefs in the supernatural, mysticism, prophecy, etc.) [26] A British study found that more than half of their study sample expressed different degrees of endorsement for conspiracy theories related to the vaccine, and specific or general coronavirus conspiracy beliefs are associated with lower self-reported vaccination compliance [27]. Therefore, the impact of conspiracy theories on vaccination rates is determined by the public’s degrees of endorsement of conspiracy theories. Besides, the concerns of a coronavirus conspiracy were also associated with generalized mistrust of the government and the healthcare system. Thus, how governments or health institutions implement vaccine interventions, such as science introductions for non-religious groups and communication with religious leaders, such as being respectful of religious beliefs, are critical to effectively reducing the transmission of conspiracy beliefs or anti-vaccine sentiment in both non-religious and main religious groups.

*Political Affiliation*. Another important finding of our research is that democratic party participants had significantly higher vaccination compliance compared to Independents and Republicans (Independents: OR: 0.2, 95% CI: 0.1, 0.4; OR: 0.3, 95% CI: 0.2 0.7). This finding is consistent with the study by Don et al. and Carolyn et al., which found decreased engagement in preventive health behaviors and vaccination intention in regions with a high proportion of Republican voters compared to regions with Democrat voters, and this difference in vaccination intentions between political parties has grown over time, with Democrats remaining stable and Republicans declining [28,29,30]. According to innovation theory, before deciding whether to adopt or reject the innovation, people will go through two stages: gaining knowledge about the innovation and getting persuaded about the innovation. Political affiliation serves as a valuable and influential social label that subliminally influences people’s health behaviors through media campaigns in these two stages. A longitudinal study demonstrated that the polarization of threat perceptions of the pandemic could be explained by the influence of social media [30]. For instance, Twitter, one of the world’s most influential social media platforms, has become a venue for political parties to issue remarks with significant influence. Goran et al. analyzed the political intent of the accounts through a collection of 137 million anti-vaccine tweets and found that most accounts were right-leaning. In addition, these accounts used reports of rare vaccine side effects from medical journals to transmit inappropriate vaccine impressions to the public [31]. Moreover, Fox News was the most popular news source among Republicans, while Democrats preferred CNN [32]. Based on this finding, a Pew Research Center poll reported that 56% of Fox News viewers think the media overstated the Coronavirus epidemic, compared to 25% of CNN viewers [33]. This exaggerated media coverage has a significant impact on the public’s trust in the media and how seriously they regard threats [34]. On the other hand, the media audience perceived the media coverage of the pandemic as inaccurate and exaggerated, proving their true perception of the severity of the pandemic.

### Limitations

There are limitations since this survey was conducted online using a convenience sample. Even though there are important regional differences in pandemic policies and epidemiological burden by state, we were unable to examine state-specific differences given the smaller sample size. Even though we removed respondents who completed the survey in a short amount of time, there is still a possibility that some respondents who were less honest or focused than others were included in the analysis.

Further cross-sectional or longitudinal studies are needed to confirm and corroborate the findings of this research, particularly given small cell sizes.

## 5. Conclusions

The study examined the variance of COVID-19 vaccination compliance in US adults in August 2022 by demographic characteristics. We found that race/ethnicity, religion, and political affiliation are highly associated with vaccination compliance. Non-religion, non-democrats, and NH black Americans had relatively low vaccine adherence. In order to decrease these demographic disparities and enhance people’s awareness of public health, the government needs to establish a positive image by releasing impartial messages through the media. For public health professionals, it is important to recognize vaccine hesitancy and conduct effective vaccination interventions regarding low vaccination compliance groups.

## Figures and Tables

**Table 1:** Vaccination completeness, and its distribution by demographic characteristics, race/ethnicity, religion, and political affiliation, stratified by vaccination completeness, United States, August 2022.

		Overall	Unvaccinated (row %)	Partially Vaccinated (row %)	Completed Primary Series (row %)	Received ≥1 Booster Dose (row %)	*p*-Value
Series Completion (row %)		700 (100%)	127 (17%)	45 (5%)	130 (15%)	385 (63%)	
Gender	Male	329 (48%)	44 (13%)	24 (6%)	55 (15%)	206 (66%)	0.0076
	Female	355 (52%)	83 (22%)	21 (4%)	73 (14%)	178 (60%)	
	Other	3 (0.2%)	0	0	2 (80%)	1 (20%)	
Age	18–24	182 (12%)	44 (27%)	13 (9%)	49 (24%)	76 (40%)	<0.0001
	25–34	183 (18%)	33 (19%)	15 (9%)	39 (21%)	96 (51%)	
	35–44	195 (17%)	30 (14%)	16 (12%)	29 (13%)	120 (61%)	
	≥45	127 (53%)	20 (16%)	1 (1%)	13 (11%)	93 (73%)	
Region	US Midwest	117 (22%)	25 (19%)	10 (6%)	18 (9%)	64 (67%)	0.1660
	US Northeast	156 (20%)	19 (12%)	5 (2%)	29 (20%)	103 (66%)	
	US South	275 (36%)	62 (22%)	18 (5%)	52 (13%)	143 (60%)	
	US West	139 (23%)	21 (14%)	12 (7%)	31 (18%)	75 (61%)	
Education	High school or Lower	197 (25%)	61 (27%)	24 (13%)	29 (11%)	83 (49%)	<0.0001
	Associate’s degree	174 (25%)	39 (25%)	3 (1%)	47 (17%)	85 (56%)	
	Bachelor’s degree	201 (30%)	14 (4%)	11 (3%)	36 (13%)	140 (79%)	
	Master’s or Higher	115 (21%)	13 (15%)	7 (4%)	18 (16%)	77 (64%)	
Race/Ethnicity	Other	45 (8%)	5 (11%)	2 (3%)	14 (21%)	24 (65%)	<0.0001
	Non-Hispanic Black	82 (11%)	32 (41%)	5 (6%)	15 (18%)	30 (35%)	
	Hispanic	113 (15%)	16 (10%)	14 (13%)	25 (25%)	58 (52%)	
	Non-Hispanic White	447 (66%)	74 (16%)	24 (3%)	76 (11%)	273 (70%)	
Religion	Catholic/Orthodox	97 (19%)	13 (11%)	4 (2%)	21 (19%)	59 (68%)	<0.0001
	Evangelical	171 (25%)	23 (13%)	15 (8%)	31 (18%)	102 (60%)	
	Jewish	28 (5%)	2 (10%)	1 (1%)	4 (12%)	21 (76%)	
	Mainline	44 (10%)	7 (7%)	1 (4%)	7 (4%)	29 (85%)	
	Muslim	42 (4%)	0	6 (17%)	3 (5%)	33 (77%)	
	Nothing	220 (29%)	53 (28%)	14 (4%)	52 (15%)	101 (52%)	
	Other	58 (6%)	21 (34%)	2 (5%)	8 (10%)	27 (52%)	
	Other Christian	27 (3%)	8 (21%)	2 (4%)	4 (8%)	13 (67%)	
Political Affiliation	Democrat	312 (41%)	35 (11%)	16 (4%)	56 (15%)	205 (71%)	0.0018
	Independent	191 (29%)	62 (27%)	8 (3%)	39 (13%)	82 (56%)	
	Republican	184 (30%)	30 (16%)	21 (9%)	35 (16%)	98 (59%)	

**Table 2:** Vaccination timing, and its distribution by demographic characteristics, race/ethnicity, religion, and political affiliation, United States, August 2022.

		Unvaccinated (row %)	Unprovided (row %)	Started in 2020 and Early 2021 (row %)	Started Late 2021 (row %)	Started in 2022 (row %)	*p*-Value
Overall (row %)		127 (17%)	35 (4%)	423 (65%)	94 (11%)	21 (2%)	
Gender	Male	44 (12%)	18 (5%)	212 (69%)	50 (10%)	11 (3%)	0.0241
	Female	83 (22%)	16 (4%)	209 (61%)	44 (12%)	10 (2%)	
	Other	0	1 (34%)	2 (66%)	0	0	
Age	18–24	44 (26%)	16 (9%)	93 (47%)	29 (16%)	3 (2%)	<0.0001
	25–34	33 (18%)	6 (4%)	114 (61%)	23 (11%)	11 (6%)	
	35–44	30 (14%)	8 (4%)	123 (60%)	33 (18%)	6 (4%)	
	≥45	20 (16%)	5 (4%)	93 (72%)	9 (7%)	1 (1%)	
Region	US Midwest	25 (18%)	8 (5%)	66 (59%)	15 (14%)	5 (4%)	0.0879
	US Northeast	19 (12%)	10 (5%)	103 (70%)	24 (11%)	2 (1%)	
	US South	62 (21%)	11 (5%)	167 (63%)	32 (8%)	8 (2%)	
	US West	21 (14%)	6 (2%)	87 (70%)	23 (12%)	6 (3%)	
Education	High school or Lower	61 (26%)	12 (8%)	96 (52%)	24 (11%)	8 (3%)	<0.0001
	Associate’s degree	39 (24%)	14 (6%)	106 (60%)	18 (9%)	2 (1%)	
	Bachelor’s degree	14 (4%)	7 (3%)	140 (74%)	33 (14%)	9 (5%)	
	Master’s or Higher	13 (15%)	2 (1%)	81 (74%)	19 (9%)	2 (1%)	
Race/Ethnicity	Other	5 (11%)	2 (3%)	30 (70%)	7 (14%)	1 (1%)	<0.0001
	Non-Hispanic Black	32 (39%)	5 (5%)	38 (44%)	8 (8%)	3 (3%)	
	Hispanic	16 (10%)	7 (7%)	66 (62%)	18 (14%)	9 (7%)	
	Non-Hispanic White	74 (16%)	21 (4%)	289 (69%)	61 (10%)	8 (1%)	
Religion	Catholic/Orthodox	13 (11%)	3 (3%)	69 (77%)	13 (8%)	1 (1%)	<0.0001
	Evangelical	23 (13%)	5 (5%)	107 (62%)	31 (16%)	8 (3%)	
	Jewish	2 (10%)	2 (8%)	21 (75%)	4 (7%)	0	
	Mainline	7 (7%)	1 (1%)	29 (80%)	6 (12%)	1 (1%)	
	Muslim	0	0	37 (80%)	6 (20%)	0	
	Nothing	53 (28%)	15 (4%)	124 (58%)	24 (8%)	7 (4%)	
	Other	21 (33%)	8 (16%)	21 (34%)	8 (13%)	2 (3%)	
	Other Christian	8 (20%)	1 (2%)	15 (67%)	2 (4%)	2 (7%)	
Political Affiliation	Democrat	35 (11%)	16 (6%)	215 (72%)	41 (9%)	9 (2%)	<0.0001
	Independent	62 (27%)	11 (4%)	93 (54%)	27 (14%)	4 (1%)	
	Republican	30 (16%)	8 (3%)	115 (67%)	26 (10%)	8 (4%)	

**Table 3:** Compliance to COVID-19 vaccination guidelines in multinomial, multivariable logistic regression models, United States, August 2022.

		Medium Compliance ^a^ OR (95% CI)	High Compliance ^b^ OR (95% CI)	*p*-Value
Overall (row %)		184 (22%)	389 (61%)	
Gender	Male	REF	REF	0.0245
	Not male	0.4 (0.2, 0.8)	0.5 (0.3, 0.9)	
Age	18–24	1.3 (0.5, 3.0)	1.1 (0.5, 2.4)	0.2278
	25–34	REF	REF	
	35–44	1.0 (0.5, 2.2)	1.1 (0.5, 2.3)	
	≥45	0.5 (0.2, 1.4)	1.3 (0.6, 2.8)	
Region	US Midwest	1.4 (0.5, 4.4)	1.1 (0.4, 3.0)	0.1433
	US Northeast	1.3 (0.4, 3.9)	0.5 (0.2, 1.4)	
	US South	0.5 (0.2, 1.5)	0.6 (0.3, 1.5)	
	US West	REF	REF	
Education	High school or Lower	0.2 (0.1, 0.5)	0.2 (0.1, 0.4)	0.0010
	Associate’s degree	0.1 (0.1, 0.4)	0.2 (0.1, 0.5)	
	Bachelor’s degree	REF	REF	
	Master’s or Higher	0.2 (0.0, 0.5)	0.3 (0.1, 0.8)	
Race/Ethnicity	Other	1.8 (0.4, 8.1)	1.4 (0.3, 5.6)	0.1433
	Non-Hispanic Black	0.3 (0.1, 0.9)	0.2 (0.1, 0.5)	
	Hispanic	3.1 (1.3, 7.0)	1.3 (0.6, 2.7)	
	Non-Hispanic White	REF	REF	
Religion	Catholic/Orthodox	REF	REF	0.0088
	Evangelical	1.8 (0.5, 6.3)	0.7 (0.2, 2.2)	
	Nothing	0.4 (0.1, 1.3)	0.4 (0.1, 1.1)	
	Other	1.1 (0.3, 4.1)	0.6 (0.2, 1.9)	
	Other Christian	1.5 (0.4, 5.9)	1.6 (0.6, 4.7)	
Political Affiliation	Democrat	REF	REF	<0.0001
	Independent	0.4 (0.2, 1.0)	0.2 (0.1, 0.4)	
	Republican	0.6 (0.2, 1.4)	0.3 (0.2, 0.7)	

^a^ Participants started vaccination later than June 2021, or did not receive the booster dose. ^b^ Participants started vaccination by June 2021 and received a booster dose.

## Data Availability

The data are available through: https://doi.org/10.6084/m9.figshare.21797729 (accessed on 5 February 2023).
